# Impact of a mobile phone and web program on symptom and functional outcomes for people with mild-to-moderate depression, anxiety and stress: a randomised controlled trial

**DOI:** 10.1186/1471-244X-13-312

**Published:** 2013-11-18

**Authors:** Judith Proudfoot, Janine Clarke, Mary-Rose Birch, Alexis E Whitton, Gordon Parker, Vijaya Manicavasagar, Virginia Harrison, Helen Christensen, Dusan Hadzi-Pavlovic

**Affiliations:** 1 Black Dog Institute, University of New South Wales, Hospital Road, Randwick, Sydney, New South Wales 2031, Australia; 2 School of Psychiatry, University of New South Wales, Hospital Road, Randwick, Sydney, New South Wales 2031, Australia

**Keywords:** eHealth, Public health, Depression, Anxiety, Psychological stress, Mobile health, Intervention studies, Work functioning

## Abstract

**Background:**

Mobile phone-based psychological interventions enable real time self-monitoring and self-management, and large-scale dissemination. However, few studies have focussed on mild-to-moderate symptoms where public health need is greatest, and none have targeted work and social functioning. This study reports outcomes of a CONSORT-compliant randomised controlled trial (RCT) to evaluate the efficacy of *myCompass*, a self-guided psychological treatment delivered via mobile phone and computer, designed to reduce mild-to-moderate depression, anxiety and stress, and improve work and social functioning.

**Method:**

Community-based volunteers with mild-to-moderate depression, anxiety and/or stress (*N* = 720) were randomly assigned to the myCompass program, an attention control intervention, or to a waitlist condition for seven weeks. The interventions were fully automated, without any human input or guidance. Participants’ symptoms and functioning were assessed at baseline, post-intervention and 3-month follow-up, using the Depression, Anxiety and Stress Scale and the Work and Social Adjustment Scale.

**Results:**

Retention rates at post-intervention and follow-up for the study sample were 72.1% (*n* = 449) and 48.6% (*n* = 350) respectively. The myCompass group showed significantly greater improvement in symptoms of depression, anxiety and stress and in work and social functioning relative to both control conditions at the end of the 7-week intervention phase (between-group effect sizes ranged from *d* = .22 to *d* = .55 based on the observed means). Symptom scores remained at near normal levels at 3-month follow-up. Participants in the attention control condition showed gradual symptom improvement during the post-intervention phase and their scores did not differ from the myCompass group at 3-month follow-up.

**Conclusions:**

The myCompass program is an effective public health program, facilitating rapid improvements in symptoms and in work and social functioning for individuals with mild-to-moderate mental health problems.

**Trial registration:**

Australian New Zealand Clinical Trials Registry ACTRN 12610000625077

## Background

Anxiety and depressive disorders are common mental health conditions with global lifetime prevalence rates of 28.8% and 16.6% respectively [1]. Both are associated with substantial impairment [2,3]. Public health interventions are needed for individuals with mild-to-moderate symptoms to reduce the risk of work and social disability, loss of quality of life, symptom exacerbation and co-existing health complications, as well as to mitigate economic consequences including increased health costs and reduced work productivity [4,5]. Decrements in work productivity due to depression alone costs an estimated US $33 billion annually in lost revenue and accounts for the largest proportion of the personal financial burden on sufferers [5]. However, the majority of people with common mental health conditions do not access professional help, despite the existence of effective psychological and pharmacological treatments [6]. Reasons include lack of service availability (especially in rural and remote areas), problems recognising symptoms, treatment cost, time constraints and concerns about confidentiality and stigma [7,8].

Web-based psychological interventions can facilitate access to empirically supported treatments, and are popular with users [9], cost-effective [10] and clinically efficacious, with effect sizes similar to face-to-face therapy [11]. Whereas these interventions have predominantly been available on desktop computers, mobile phones offer several further advantages. Practically, their wide-spread ownership (currently 6 billion subscriptions worldwide [12]), multiple functionalities including internet linkage, and the fact that they are generally carried on the person and turned on, makes mobile phones an ideal platform for broad dissemination of effective psychotherapies. Clinically, mobile phones have the capacity to support ecological momentary assessment and ecological momentary intervention (EMA and EMI); that is, self-monitoring of symptoms and behaviours and provision of psychotherapeutic advice in real-time and in real-world contexts [13].

The number of mobile mental health interventions is increasing rapidly, although research into their efficacy is not occurring at the same pace [13]. Predominantly, extant research has been confined to small, non-controlled, and non-randomised studies without long-term follow-up [14], with only a few exceptions [15,16]. Furthermore, none of the studies has tested the effects of mobile interventions on work and social functioning, where the personal and socio-economic burden is great.

We have previously reported data from a proof of concept study suggesting the feasibility of an intervention that combines web and mobile technologies for improving psychological well being [14]. The myCompass program (http://www.myCompass.org.au) is an automatic, self-guided, public health program aimed at facilitating self-management of common mental health problems. Grounded in cognitive behavioural therapy (CBT), and incorporating elements of Problem Solving Therapy, Interpersonal Psychotherapy and Positive Psychology, the myCompass program combines round-the-clock availability of psychotherapeutic resources with EMA (via mobile phone and computer). In the quasi-experimental study, 49 people with mild-to-moderate mental health symptoms showed significant improvements in depression, anxiety and stress and overall psychological distress after using the program for six weeks. Furthermore, a high level of satisfaction with the myCompass intervention was reported [14].

The aim of this paper is to report the outcomes of a CONSORT-compliant randomised controlled trial (RCT) to evaluate the efficacy of the myCompass program in a large community sample of people experiencing mild-to-moderate depression, anxiety and/or stress. We predicted that symptoms of depression, anxiety and stress would reduce in participants randomly allocated to receive myCompass, relative to both attention control (AC) and waitlist (WL) conditions. We also predicted that use of myCompass would increase work and social functioning relative to the AC and WL conditions. To our knowledge, this is the first trial to examine a fully automated, self-help, mobile phone and web-based intervention for common mental health problems and work and social functioning in a community sample.

## Methods

### Eligibility criteria

Participants were required to meet the following criteria: Australian resident aged 18 to 75 years; own an internet-enabled mobile phone; have access to a desk-top computer with internet capability; have a valid email address; and report symptoms of mild-to-moderate depression, anxiety and/or stress, defined as a total score of 27–63 inclusive on the Depression Anxiety and Stress Scales (DASS; [17]). Consistent with our public health focus on mild-to-moderate symptoms, people were excluded if they scored 64 or more on the DASS (severe symptomatology), answered positively to questions asking about suicidal thoughts, intent and/or previous suicide attempts, or met criteria for psychotic symptoms, as measured by the Psychosis Screening Questionnaire (PSQ; [18]).

### Sample & setting

Based on data from our proof of concept study [14], our targeting of mild-to-moderate symptoms, and the fact that myCompass is a self-help intervention without therapist support, we estimated a between-group effect size of *d* = 0.2. Power calculations established that for 80% power and an alpha of .05, 400 people per group would be required to detect a difference if one was present. To allow for sample attrition, we set a sample size target of *n* = 650 per group. Participants were recruited nationally and online between October 2011 and March 2012 via social media (Facebook and Twitter), websites of the Black Dog Institute (BDI), corporate and government organisations, advertisements placed on national radio and in print media, and the BDI volunteer research register. Due to time and funding restrictions, it was not possible to extend recruitment activities beyond this period. Written informed consent was provided by all participants. The RCT was approved by the Human Research Ethics Committee at the University of New South Wales, Sydney, Australia (HREC 100019), and registered as Australian New Zealand Clinical Trials Registry ACTRN 12610000625077.

### Design

A mixed factorial repeated measures design was employed, with full randomisation to three conditions.

### Randomisation

A research assistant not involved in the RCT randomised participants after baseline using computerised random numbers. Allocation was either to the myCompass, AC or WL condition. Participants received advice of their group assignment by email.

### Interventions

myCompass is a fully-automated, self-help, public health intervention, that is tailored to the user and has no therapist input. Real-time self-monitoring of symptoms (e.g., problem moods, thoughts and behaviours) via mobile phone and/or computer is a key therapeutic feature. Users can self-monitor three symptoms of their choice at any one time, selected from a list of 20, or three that are recommended to them by the program (e.g., confidence, worry, irritability, motivation, diet, and medication use). Each symptom is rated on a 10-point scale (e.g., “*how confident do you feel right now*”, “*how worried do you feel right now*”, “*how satisfied are you that you have taken your prescribed medication today*”). At the time of rating, users also provide contextual information about where they are, what they are doing and who they are with, using a series of drop-down menus. Users can schedule short message service (SMS) or email reminders to facilitate self-monitoring (frequency of reminders determined by the user); receive and print graphical feedback about their monitoring, including contextual information, on their phone or computer (to monitor change and assist identification of triggers); and elect to receive helpful facts, mental health-care tips or motivational statements by SMS or email.

Evidence-based and interactive psychological modules that users can complete via the internet on their computers are another key element of myCompass. The program contains 12 skill-building modules derived from CBT, Interpersonal Psychotherapy, Problem-solving Therapy and Positive Psychology that cover topics such as Managing Fear and Anxiety, Tackling Unhelpful Thinking, Managing Loss and Major Life Change, and Solving Problems. Each module comprises three 10-minute sessions and includes activities for users to complete on the computer. Modules also recommend home practice tasks for completion between the weekly sessions to promote skill generalisation. Users can complete the modules of their choice, or those recommended to them by the myCompass program.

Tailoring of the myCompass program self-monitoring and module recommendations occurs via users’ responses to a profile questionnaire completed at registration. This questionnaire asks users to rate the extent to which they experience a range of symptoms of depression, anxiety, and stress (e.g., worry, irritability, difficulties with motivation and concentration). Targeting the three highest rated symptoms, in-built algorithms generate personalised feedback to the user about the self-monitoring dimensions and psychological modules that may be of greatest benefit (i.e., each dimension and module is weighted according to its clinical relevance for each symptom, and those with the highest weightings are recommended).

User privacy is managed by a password protected log-on, and by ensuring that user generated data (i.e., self-monitoring ratings) are not stored on the users’ phone but instead transferred via the internet using secure sockets layer protocols (which encrypt transmitted data by rendering it unreadable to anyone other than the intended recipient) and by storing the data in secure servers. Registering to use myCompass is free and users are not billed for the SMSs they receive.

Participants randomised to the myCompass intervention were emailed instructions for accessing and logging onto the program. They were advised that they had full access to the program for seven weeks, and were encouraged to use the program *ad libitum* during this time. However, it was recommended that they complete a minimum of two modules and monitor daily at least three moods or behaviours during the intervention phase. Access to the program was withdrawn at the end of seven weeks.

*Attention control participants* received a control mental health program matched to the active intervention on duration and mode of delivery. Each week for seven weeks*,* they received a fact sheet containing information about depression, anxiety or stress sent to their email address. The information was designed to be read on computer in approximately 10 minutes, and to be credible but void of management advice or treatment strategies. They also received on their mobile phones weekly SMS messages containing brief factual statements about depression, anxiety and stress. The mobile phone statements were also therapeutically inactive, but chosen to ensure that the control program had face validity. Messages varied in length from 160 to 275 characters so as to match, as far as possible, the minimum amount of SMS content that myCompass participants received each week, namely one self-monitoring reminder (160 characters) plus an optional motivational statement, mental health care tip or fact (average length 95 characters).

*Waitlist participants* did not receive emails or SMSs during the intervention phase, but received full access to the myCompass program at the end of the seven weeks.

By including both the AC group and the WL group, we were able to control for non-specific effects of the intervention. We were also able to test whether any effects seen during the intervention phase were replicated when the WL control group subsequently completed the myCompass program.

### Procedure

Data collection took place between October 2011 and October 2012. All study consent, screening and questionnaire data were collected online using online survey software. Individuals not meeting screening criteria received automated feedback explaining the reason/s for their ineligibility, and referring them to other online resources of the BDI. Individuals with symptoms in the severe range were also advised to seek face-to-face support from a health professional and were provided the contact details of crisis support services.

Eligible participants completed a baseline questionnaire prior to randomisation, a post-intervention questionnaire administered at eight weeks, and a follow-up questionnaire administered 12 weeks later for participants in the myCompass and AC groups, and 19 weeks later for the WL group. At each assessment point, participants accessed the appropriate study questionnaire via a link sent to them in an email message.

### Primary outcome

*The Depression, Anxiety and Stress Scales* measure (DASS-21; [17]) is a widely used self-report measure of depression, anxiety and stress with high internal consistency, acceptable test-retest reliability [19], and yields reliable and valid data when used in an online format [20]. Respondents are asked to indicate the frequency with which they experienced symptoms of depression, anxiety and stress over the previous week. Total scores range from 0 to 126 and subscale scores range from 0 to 42, with higher scores indicating greater symptom severity.

### Secondary outcomes

*The Work and Social Adjustment Scale* (WSAS; [21]) assesses the degree to which mental health problems interfere with day-to-day functioning in five domains: work, social leisure activities, private leisure activities, home-management, and personal relationships [21]. The measure provides an assessment of the experiential impact of mental health symptoms from the sufferer’s point of view. Scores range from 0 to 40, with higher scores indicating poorer adjustment. Meyer et al. [22] provide data supporting the psychometric adequacy of the WSAS when administered in an online format.

Demographic and clinical information collected within the baseline questionnaire included age, gender, marital status, educational level, employment status, frequency of mobile phone and internet use, frequency of depressive and anxiety episodes, and age of first episode. At each assessment point, health service utilisation for mental health reasons (e.g., visits to general practitioner, mental health professionals and alternative health practitioners) and use of antidepressant and anxiolytic medication were also assessed. Satisfaction with the program was measured at post-intervention for participants in the myCompass and AC groups using a study-specific measure assessing users’ perceptions of program usability, content, flexibility and functionality.

Adherence, defined as the extent to which participants engaged with the intervention [23], was examined for the myCompass group with respect to three indices, namely, frequency of logins, frequency of self-monitoring, and number of modules attempted.

### Statistical analyses

Statistical analyses were completed with SPSS 20.0 software. Characteristics of the three groups at baseline were compared using chi-square tests for categorical variables and analysis of variance (ANOVA) for continuous variables. Chi-square and ANOVA were also used to compare baseline characteristics of participants who did (‘non-dropouts’) and did not (‘dropouts’) return completed questionnaires at post-intervention and follow-up, to explore possible biases in attrition.

Effects of the myCompass intervention on study outcomes were evaluated using intention-to-treat (ITT) analyses that included data from all participants who completed the baseline assessment and any follow-up assessment. Strategies for dealing with missing data in longitudinal studies vary, so we adopted two recommended techniques for analysing incomplete datasets, namely, mixed models repeated measures (MMRM) and multiple imputation (MI; for a review see [24]). The two analyses yielded similar outcomes and, as MMRM is the dominant strategy adopted in studies of web-based interventions, the findings derived from MMRM are presented here.

In MMRM, no participant is removed from the analysis because all available data is used to obtain parameter estimates. In the present study, restricted maximum likelihood (REML) was used to estimate model parameters and error degrees of freedom were calculated using Satterthwaite’s approximation [25]. Analyses assumed a compound symmetric structure, in line with Fairclough’s recommendation that the covariance structure be restricted in situations where attrition is high [26].

Post-intervention analyses involved a series of 3 groups (myCompass, AC and WL) by 2 times (pre-intervention, post-intervention) repeated measures models. Because WL participants were exposed to the myCompass intervention during the follow-up period, repeated measures models analysing follow-up data comprised 2 groups (myCompass and AC) by 3 times (pre, post and follow-up). For each analysis, scores at baseline on the outcome variable of interest was entered as a covariate. Significant group by time interaction effects were examined using sets of Bonferroni adjusted interaction contrasts to compare between-group differences in mean change on the outcome measure from baseline to post-intervention (i.e., intervention period) and from post-intervention to follow-up (i.e., follow-up phase), as appropriate. All effects were tested at the *p* < 0.05 level, with adjustment according to the number of contrasts in each set.

Cohen’s *d* was calculated following the procedure outlined in Ruwaard et al. [27] and using both observed and estimated marginal means. For all outcome measures, within-and between-group differences were standardised to Cohen’s *d* using the pooled standard deviation of the observed scores obtained at baseline.

To determine the replicability of treatment effects resulting from the intervention, primary and secondary outcome data from WL participants who used the myCompass program at the end of their waiting period were examined at post-intervention and follow-up using paired samples *t*-tests and Cohen’s *d.*

## Results

### Randomisation and study attrition

**Figure 1:**
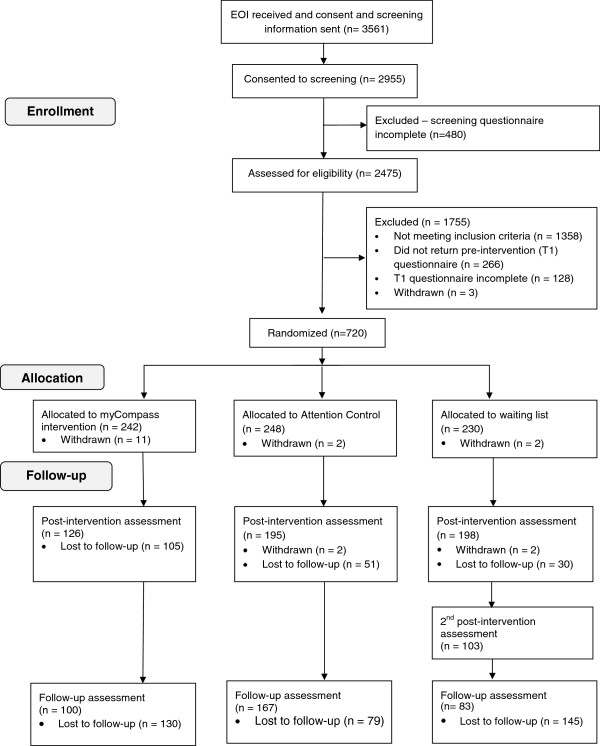
Participant flow diagram.

Details of enrolment into the trial, organised according to the CONSORT guidelines [28], are shown in Figure 1. Of the 3561 people who expressed an interest in the study, 720 were randomised. Among those who returned completed screening information, reasons for ineligibility included: symptoms too severe (975, 73.1%); no internet-enabled mobile phone (167, 12.3%); symptoms in the normal range (150, 11%); no access to the internet or mobile phone (49, 3.6%) and not an Australian resident (17, 0.01%). Fifteen people subsequently withdrew from the study and, in the absence of consent to use their data, we excluded them from the analyses.

The recruitment process yielded a sample of *N* = 720 participants. The sample was predominantly female (491, 69.6%), university educated (387, 53.7%), employed (591, 83.8%), married (288, 41%), and with a mean age of 38.9 years. The three groups did not differ on the demographic and clinical history variables assessed at baseline, with two exceptions: WL participants (66%) were more likely than myCompass (58%) and AC (52%) participants to report a stressful episode in the month preceding the study (*X*^2^ = 8.08, *P* < 0.02), and had a significantly lower score at baseline on the DASS Anxiety subscale than myCompass participants (*F*_2_, _702_ = 4.44, *P* = 0.01; Table 1).

**Table 1:** Demographic and clinical characteristics of the intervention, attention control (AC) and waitlist (WL) groups at baseline

**Characteristics**	**myCompass**	**Attention control**	**Wait list**
	**(n = 231)**	**(n = 246)**	**(n = 228)**
*Socio-demographics*			
Mean age (SD)	39 (10.73)	40 (11.42)	38 (10.26)
Female	160 (70%)	170 (70%)	161(70%)
Married	97 (42%)	104 (42%)	87 (38%)
Employed	196 (85%)	206 (84%)	189 (83%)
Student	46 (20%)	53 (22%)	47 (21%)
*Education level*			
Secondary school or lower	25 (11%)	47 (19%)	41 (18%)
Trade certificate or diploma	70 (30%)	79 (32%)	50 (22%)
University under-graduate or more	135 (58%)	118 (48%)	134 (59%)
Daily computer use	225 (97%)	234 (95%)	219 (96%)
Daily mobile phone use	223 (96%)	234 (95%)	220 (96%)
*Clinical history*			
*Experienced an episode*			
Depression	201 (87%)	222 (90%)	198 (87%)
Anxiety	188 (81%)	208 (85%)	196 (86%)
Stress	221 (96%)	241 (98%)	225 (99%)
*Frequency of previous episodes*			
Depression - > 5 times	116 (50%)	109 (44%)	106 (46%)
Anxiety - > 5 times	106 (46%)	107 (43%)	108 (47%)
Stress - > 5 times	153 (66%)	146 (59%)	132 (58%)
*Episode in the last month*			
Depression	82 (35%)	108 (44%)	89 (39%)
Anxiety	68 (29%)	87 (35%)	87 (38%)
Stress	134 (58%)	129 (52%)	151 (66%)
*Distress caused by most recent episode (Mean, SD)*^*a*^			
Depression	7.98 (1.78)	7.75 (2.25)	8.09 (2.06)
Anxiety	7.82 (1.67)	7.44 (1.92)	8.02 (1.89)
Stress	7.64 (1.75)	7.54 (2.07)	7.52 (2.08)
Comorbid symptoms^b^	140 (61%)	161 (65%)	149 (65%)

^a^Scores ranged 0 (No distress) to 10 (Extremely distressing).

^b^Scores in the mild range or higher on two or more of the DASS subscales.

The rate of attrition for the total sample was 27.9% at post-intervention and 51.4% at the 3-month follow-up assessment (see Figure 1). At post-intervention, attrition was higher among employed (27.9%) than unemployed participants (18.4%; *X*^2^_1_ = 4.04, *P* = 0.04), slightly higher in males (31.6%) than females (24.4%; *X*^2^_1_ = 3.77, *P* = 0 .05) and higher in the myCompass group (45.5%) than the AC (20.7%) and WL groups (13.2%; *X*^2^_2_ = 67.84, *P* < 0.001). Those who did not complete the post-intervention assessment also reported significantly lower DASS Anxiety scores at baseline (*t*_703_ = -1.96, *P* = 0.05). Attrition rates at 3-month follow-up were 56.7% and 32.1%, for the myCompass and AC groups respectively, and differed significantly (*X*^2^_1_ = 29.25, *P* < 0.001). Demographic variables did not differ between dropouts and non-dropouts at follow-up, however, dropouts reported significantly less functional impairment at baseline on the WSAS (*t*_703_ = -2.01, *P* = 0.04).

For the myCompass group, there were no significant baseline differences between non-dropouts and dropouts at post-intervention and follow-up on any of the demographic and clinical history variables.

### Outcomes at post-intervention

Table 2 reports observed and estimated marginal mean (EMM) scores on the study outcomes at baseline, post-intervention and follow-up for the three groups.

**Table 2:** Observed and estimated marginal means (EMM), including SD and SEM, at pre-and post-intervention and follow-up for each group

	**myCompass**		**Attention control**		**Wait list**^**a**^	
n’s at each time-point						
Baseline	231		246		228	
Post-intervention	126		195		198	
Follow-up	100		167		-	
Outcome	Mean *(SD)*	EMM *(SEM)*	Mean *(SD)*	EMM *(SEM)*	Mean *(SD)*	EMM *(SEM)*
***WSAS***						
Baseline	16.78 *(8.07)*	16.79 *(0.29)*	16.69 *(8.11)*	16.73 *(0.28)*	16.56 *(8.77)*	16.79 *(0.29)*
Post-intervention	12.63 *(8.30)*	12.26 *(0.41)*	14.42 *(9.47)*	14.41 *(0.32)*	15.03 *(8.97)*	15.05 *(0.32)*
Follow-up	12.02 *(8.83)*	12.13 *(0.54)*	13.26 *(9.05)*	13.27 *(0.42)*	-	
***DASS depression***						
Baseline	15.09 *(9.44)*	14.98 *(0.34)*	15.10 *(8.88)*	14.98 *(0.33)*	14.83 *(8.90)*	14.98 *(0.34)*
Post-intervention	10.62 *(8.41)*	10.57 *(0.47)*	13.84 *(10.41)*	13.82 *(0.37)*	14.82 *(9.96)*	14.95 *(0.37)*
Follow-up	11.68 *(9.86)*	12.14 *(0.61)*	12.59 *(9.53)*	12.51 *(0.47)*	-	
***DASS anxiety***						
Baseline^b^	7.31 *(6.47)*	8.55 *(0.27)*	8.83 *(7.05)*	8.55 *(0.26)*	9.09 *(7.27)*	8.55 *(0.27)*
Post-intervention	5.63 *(6.74)*	6.40 *(0.37)*	8.43 *(8.18)*	8.17 *(0.29)*	8.91 *(7.98)*	8.38 *(0.29)*
Follow-up	6.78 *(7.31)*	7.00 *(0.46)*	7.43 *(7.41)*	7.15 *(0.36)*	-	
***DASS stress***						
Baseline	18.12 *(8.02)*	18.45 *(0.32)*	18.46 *(8.72)*	18.45 *(0.31)*	18.67 *(7.91)*	18.45 *(0.32)*
Post-intervention	14.94 *(7.62)*	15.17 *(0.43)*	16.73 *(8.95)*	16.76 *(0.34)*	17.82 *(8.96)*	17.47 *(0.34)*
Follow-up	14.26 *(8.37)*	14.66 *(0.56)*	16.00 *(9.18)*	15.84 *(0.44)*	-	
***DASS total score***						
Baseline	40.53 *(18.94)*	41.99 *(0.79)*	42.40 *(19.53)*	41.99 *(0.76)*	42.60 *(18.45)*	41.99 *(0.79)*
Post-intervention	31.20 *(19.31)*	31.88 *(1.08)*	39.01 *(23.94)*	38.76 *(0.87)*	41.56 *(22.11)*	40.72 *(0.86)*
Follow-up	32.74 *(22.13)*	33.90 *(1.42)*	36.03 *(22.69)*	35.48 *(1.10)*	-	

^a^WL participants were given access to myCompass after the post-intervention assessment so no follow-up data are reported.

^b^WL significantly greater than intervention, *P* = .01.

The MMRM analyses of pre-post intervention data yielded significant 3 (groups) by 2 (times) interactions across the DASS subscales and total scores and the WSAS (Table 3). Bonferroni adjusted contrasts comparing change from baseline for each group showed a consistent pattern of significantly greater improvement at post-intervention for the myCompass group compared to the AC and WL groups on the Depression, Anxiety, DASS Total and WSAS scales. Compared to the WL group, myCompass participants also showed significant gains on the Stress scale.

**Table 3:** Outcomes of mixed models analyses at baseline and post-intervention for all groups

**Effect**	**Contrast estimate**	***df*** **(numerator, denominator)**	***F, t*** **statistic**^**a**^	***P***	**Confidence intervals**^**b**^
***WSAS***					
Group x time:		2, 764	9.225	.000	
Interaction contrasts:					
myCompass vs. AC^c^	2.12	1, 802	3.23	.001	0.55-3.70
myCompass vs. WL	2.77	1, 792	4.18	.000	1.18-4.36
AC vs. WL	0.65	1, 711	0.28	.286	-0.81-2.10
***DASS depression***					
Group x time		2, 757	17.16	.000	
Interaction contrasts:					
myCompass vs. AC	3.25	1, 794	4.27	.000	1.42-5.08
myCompass vs. WL	4.39	1, 779	5.72	.000	2.55-6.23
AC vs. WL	1.13	1, 709	1.59	.111	-0.57-2.83
***DASS anxiety***					
Group x time		2, 760	6.11	.002	
Interaction contrasts:					
myCompass vs. AC	1.76	1, 796	2.93	.004	0.32-3.21
myCompass vs. WL	1.96	1, 782	3.25	.001	0.51-3.42
AC vs. WL	0.20	1, 711	0.36	.714	-1.13-1.54
***DASS stress***					
Group x time		2, 758	5.33	.005	
Interaction contrasts:					
myCompass vs. AC	1.59	1, 795	2.25	.025	-0.11-3.29
myCompass vs. WL	2.29	1, 780	3.22	.001	0.50-4.00
AC vs. WL	0.71	1, 710	1.07	.283	-0.87-2.28
***DASS total score***					
Group x time		2, 758	13.065	.000	
Interaction contrasts:					
myCompass vs. AC	6.83	1, 794	3.86	.000	2.59-11.97
myCompass vs. WL	8.79	1, 780	4.95	.000	4.53-13.05
AC vs. WL	1.95	1, 709	1.19	.233	-1.93-5.90

^a^As appropriate.

^b^Bonferroni adjusted confidence intervals, *P* = .016.

^c^= ‘Attention Control’, WL = ‘Waitlist’.

### Outcomes at follow-up

**Figure 2:**
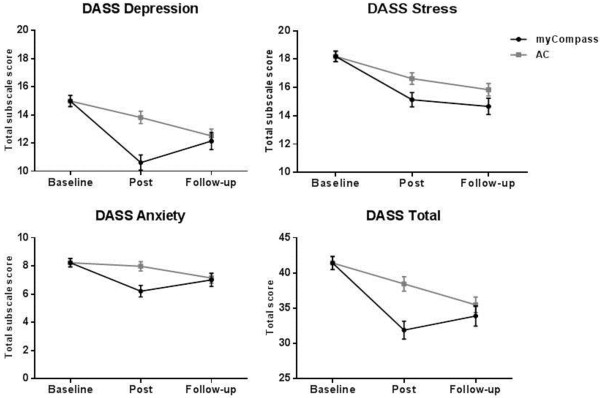
Estimated marginal DASS means for the intervention and attention control groups.

**Figure 3:**
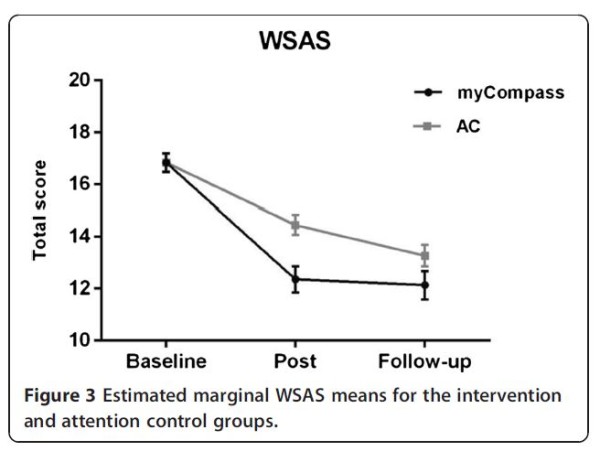
Estimated marginal WSAS means for the intervention and attention control groups.

Figures 2 and 3 depict the estimated marginal means for the myCompass and AC conditions on the primary and secondary outcome measures at baseline, post-intervention and follow-up.

When data for the myCompass and AC groups at the three time points were subjected to MMRM analysis, significant overall group by time interactions were observed across all of the primary and secondary measures, except for the DASS Stress subscale (*F*_2_, _800_ = 5.33, *P* = 0.12). In these analyses, the interaction contrasts computed for the intervention phase examined the same group differences as the contrast comparing the myCompass and AC group reported in Table 3. Interaction contrasts comparing groups during the follow-up phase showed significantly greater change for the AC group than the myCompass group on the WSAS, DASS Depression and DASS Total scales. Within-group comparisons of means in the myCompass group showed that treatment gains were maintained from post-test through the follow-up phase, with scores remaining low and stable, the only exception being a small increase in DASS Depression scores (mean difference = 1.52, *P* = 0.05) Table 4.

**Table 4:** Outcomes of mixed models analyses at baseline, post-intervention and follow-up for the myCompass and attention control groups

**Effect**	**Contrast estimate**	***df*** **(numerator, denominator)**	***F, t*** **statistic**^**a**^	***p***	**Confidence intervals**^**b**^
***WSAS***					
Group x time		2, 790	3.93	.020	
Interaction contrasts:					
Intervention phase^c^	2.06	1, 790	2.77	.006	0.39-3.73
Follow-up phase	-0.93	1, 761	-1.07	.281	-2.81-1.01
***Depression***					
Group x time		2, 803	7.94	.000	
Interaction contrasts:					
Intervention phase	3.22	1, 799	3.83	.000	1.33-5.10
Follow-up phase	-2.85	1, 774	-2.91	.004	-5.05--0.65
***DASS anxiety***					
Group x time		2, 805	4.20	.015	
Interaction contrasts:					
Intervention phase	1.76	1, 802	2.76	.006	0.33-3.21
Follow-up phase	-1.62	1, 775	-2.18	.029	-3.29--0.05
***DASS stress***					
Group x time		2, 800	5.33	.120	
Interaction contrasts:					
Intervention phase	1.48	1, 797	1.91	.056	-0.26-3.22
Follow-up phase	-0.30	1, 770	-0.33	.721	-2.33-1.72
***DASS total score***					
Group x time		2, 801	6.15	.002	
Interaction contrasts:					
Intervention phase	6.73	1, 798	3.46	.001	2.91-10.55
Follow-up phase	-5.14	1, 771	-2.27	.024	-9.59--0.69

^a^As appropriate.

^b^Bonferroni adjusted confidence intervals, *P* = .025.

^c^Although the model is different, the intervention phase contrasts answer the same question as those comparing the myCompass and Attention Control groups reported in Table 3.

### Effect size

Between- and within-group effect sizes on the primary and secondary outcomes calculated using observed and adjusted means are presented in Table 5. At post-intervention, between-group effect sizes calculated using observed means ranged from small-to-moderate (myCompass vs. AC; *d* range = .22 - .41: myCompass vs. WL; *d* range = .29 to .55), with effect sizes based on adjusted means tending to be only marginally lower. Within-group effects for the myCompass group were mostly moderate based on observed means (*d* range = .24 to .49), and slightly larger based on adjusted means. For the AC and WL groups, within-group effects were generally small (*d* range = .01 to .27).

**Table 5:** **Between and within-group effects at post-test calculated using observed and estimated marginal means (Cohen’s** ***d*)**

	**WSAS**	**DASS Subscales**			
		**Depression**	**Anxiety**	**Stress**	**Total**
**Observed means**					
*Between-groups:*					
myCompass vs. AC^a^	.22	.36	.40	.22	.41
myCompass vs. WL	.29	.46	.47	.35	.55
*Within-groups:*					
myCompass	.49	.49	.24	.39	.49
AC	.27	.13	.06	.21	.18
WL	.18	.00	.03	.10	.05
***Estimated marginal means***					
*Between-groups:*					
myCompass vs. AC	.23	.33	.26	.19	.36
myCompass vs. WL	.29	.48	.29	.28	.47
*Within-groups:*					
myCompass	.55	.49	.31	.40	.53
AC	.28	.13	.05	.21	.17
WL	.21	.00	.03	.11	.07

^a^AC = ‘Attention Control’, WL = ‘Waitlist’.

### Waitlist group results

WL participants were given access to myCompass at the end of the waiting period, and 52% (103) returned a questionnaire after using the myCompass intervention for seven weeks. Paired samples *t*-tests showed significant immediate improvement for participants on the three DASS subscales and the total DASS (*t*_101_ range = 1.97-3.10; *P* range 0.001 to 0.05) and on the WSAS (*t*_95_ = 2.94; *P* = .004). Follow-up data were provided by 66% (68) of this group and showed that these improvements were maintained at follow-up (*t*_67_ range = -1.22-1.30; NS). Within-group effect sizes calculated immediately after the intervention and using observed means were small (*d* range = .17 for DASS Anxiety to .31 for DASS Depression).

### Program adherence and satisfaction

Participants in the intervention group logged in to use the myCompass program an average of 14.7 times (*s.d.*=16.7; range 0 to 105) and self-monitored an average of 49 times (*s.d.*=54.1; range 0 to 262) during the 7-week intervention period. The mean number of modules completed was 1.6 (*s.d.*=1.7; range 0 to 9). There was no difference at baseline between people who did and did not log in to use myCompass during the intervention period. None of the adherence indices correlated with demographic, clinical history and primary and secondary outcome data obtained at baseline.

Scores on the program satisfaction measure ranged from 1 (dissatisfied) to 5 (extremely satisfied). On average, ratings of program satisfaction were above the mid-points of the scale and did not differ for participants in the myCompass (mean rating = 3.86, *s.d.=*0.82) and AC (mean rating = 3.66*, s.d.=*0.85) conditions (*t*_299_ = 1.92; NS). Eighty-three per cent of myCompass participants reported that they would recommend the program to others, and 87.4% indicated that they would happily use the program again.

## Discussion

The myCompass intervention brought about rapid improvements in mental health symptoms and in work and social functioning in a large community sample. At post-intervention, the myCompass group showed significantly reduced symptoms of depression, anxiety and stress, and significantly improved levels of work and social functioning. Mostly moderate within-group and small-to-moderate between-group effects on the primary and secondary outcome measures were found for myCompass participants. Scores reduced to the normal range by post-intervention and treatment gains were maintained at 3-month follow-up. Participants in the AC condition showed gradual improvement over the post-intervention period and no differences were observed between myCompass and AC participants at 3 month follow-up. The immediate within-group improvements seen in the myCompass group were later replicated in the WL group when they went through the myCompass intervention at the conclusion of their 7-week wait period.

These results demonstrate that delivery of CBT using a combination of mobile phone and computer technology is effective, acceptable to users and, consistent with trials of web-based interventions delivered via stationary devices, produces persistent benefits [29]. For depression, in particular, the current findings compare very favourably to effect sizes achieved in other studies of automated, unassisted web-based interventions [30].

During the follow-up phase, scores on the primary and secondary outcomes for the myCompass group remained improved, but at the 3-month follow-up no longer differed from the AC group. This is likely to reflect the continued improvement of the AC group, the complete withdrawal of the active program in the myCompass group at seven weeks, and the fact that most participants in the myCompass group achieved ‘normal’ or ‘near-normal’ levels of work and social functioning and mental health symptoms after seven weeks (i.e., scoring at ‘floor’), leaving no room for further improvement. The pattern of symptom improvement observed for the AC group is similar to the natural course of symptom remission over several months observed in untreated depression and anxiety [31,32]. In contrast, the myCompass intervention accelerated symptom remission to within two months, producing rapid benefit for those-in-need, and with effect sizes predominantly in the moderate range. Work and social functioning also significantly improved within two months in the myCompass group, indicating that the program positively impacts on the personal burden associated with anxiety and mood disorders. These results were achieved despite the smaller than planned final sample size, and suggest a larger therapeutic benefit for myCompass than was anticipated in the initial study design.

### Limitations

Limitations of this research need to be acknowledged. In common with previous internet trials [23,33], dropout attrition was high, especially for the myCompass group, and rates of engagement for myCompass participants with the program content were highly variable (and in some instances minimal). Inspection of possible biases due to attrition showed that dropouts were more likely to be male and employed, thus reducing our confidence in generalising to these groups. While our statistical methods accounted for dropout attrition and non-completion, we cannot discount the possibility that dropouts from the intervention group were less satisfied with the program and/or experienced less positive outcomes. Further research is required to examine the predictors of usage of the myCompass program, and relations between program usage and symptom and functional gains.

To the best of our knowledge, this is the first study to demonstrate the effectiveness of mobile phone and computer technology to deliver a fully automated, self-help intervention for a community sample with depression, anxiety and stress. Future research is needed to isolate the relative contributions of the mobile phone (e.g., self-monitoring, SMS messages) and computer-based (e.g., psycho-educational modules) elements of the intervention. We are currently planning a dismantling study comparing different program elements (e.g., mobile versus email self-monitoring; modules only versus modules and self-monitoring), in order to identify the ‘active’ program elements. Further research to quantify work productivity improvements and monetary savings, and to investigate the short-and long-term effectiveness of myCompass in more severely distressed individuals is also warranted.

## Conclusion

Mobile phones are a highly accessible and practical means of delivering CBT and facilitating self-monitoring for people with mild-to-moderate mental health symptoms. Given the high prevalence of functional impairment associated with mild-to-moderate anxiety and mood disorders, resource-intensive interventions are not feasible for addressing the substantial personal and economic costs associated with unmet treatment need. Mobile phone-enabled and internet-delivered interventions are accessible, popular, and capable of being disseminated at scale. myCompass is a fully automated public health intervention without therapist support that people can use via the internet on their mobile phones and computers. When used for seven weeks, the program was effective in reducing symptoms of mild-to-moderate depression, anxiety and stress, and improving work and social functioning, with improvements persisting for three months. These results are encouraging, and when the practical benefits of accessibility, anonymity and ease of widespread dissemination are considered, they suggest that the combination of mobile phone and internet technology provides an ideal platform for the delivery of mental health care. A public health intervention that is easily accessible, free to use, and capable of producing reasonable symptom gains, in the absence of therapist assistance, has the potential to be of major benefit in population terms.

## Competing interests

The authors declare that they have no competing interests.

## Authors’ contributions

JP conceived and designed the study, supervised the data collection, participated in the data analysis and interpretation of results, was involved in the drafting of the manuscript and carried out critical revision for intellectual content. JC undertook data collection, performed the statistical analysis, and drafted the manuscript; MRB undertook data collection and revised the manuscript critically; AW assisted with data collection, statistical analyses and interpretation of results, manuscript drafting and revision for intellectual content; GP was involved in the concept and design of the study, the drafting of the manuscript and revising it critically; VM participated in the concept and design of the study, the data collection, the drafting of the manuscript; VH was involved in the concept and design of the study, interpretation of results and the drafting of the manuscript; HC participated in interpretation of results, manuscript writing and revision of intellectual content; DH-P was involved in the concept and design of the study, the statistical analysis, and the drafting of the manuscript. All authors read and approved the final manuscript.

## Pre-publication history

The pre-publication history for this paper can be accessed here:

http://www.biomedcentral.com/1471-244X/13/312/prepub
